# Relationship between Oxidative Stress, ER Stress, and Inflammation in Type 2 Diabetes: The Battle Continues

**DOI:** 10.3390/jcm8091385

**Published:** 2019-09-04

**Authors:** Estefania Burgos-Morón, Zaida Abad-Jiménez, Aranzazu Martínez de Marañón, Francesca Iannantuoni, Irene Escribano-López, Sandra López-Domènech, Christian Salom, Ana Jover, Vicente Mora, Ildefonso Roldan, Eva Solá, Milagros Rocha, Víctor M. Víctor

**Affiliations:** 1 Service of Endocrinology, University Hospital Doctor Peset—Foundation for the Promotion of Health and Biomedical Research in the Valencian Region (FISABIO), 46017 Valencia, Spain; 2 Service of Cardiology, University Hospital Doctor Peset—Foundation for the Promotion of Health and Biomedical Research in the Valencian Region (FISABIO), 46017 Valencia, Spain; 3 CIBERehd - Department of Pharmacology, University of Valencia, 46010 Valencia, Spain; 4 Department of Physiology, University of Valencia, Faculty of Medicine and Odontology, 46010 Valencia, Spain

**Keywords:** antioxidants, ER stress, insulin resistance, mitochondria, oxidative stress, ROS, type 2 diabetes

## Abstract

Type 2 diabetes (T2D) is a metabolic disorder characterized by hyperglycemia and insulin resistance in which oxidative stress is thought to be a primary cause. Considering that mitochondria are the main source of ROS, we have set out to provide a general overview on how oxidative stress is generated and related to T2D. Enhanced generation of reactive oxygen species (ROS) and oxidative stress occurs in mitochondria as a consequence of an overload of glucose and oxidative phosphorylation. Endoplasmic reticulum (ER) stress plays an important role in oxidative stress, as it is also a source of ROS. The tight interconnection between both organelles through mitochondrial-associated membranes (MAMs) means that the ROS generated in mitochondria promote ER stress. Therefore, a state of stress and mitochondrial dysfunction are consequences of this vicious cycle. The implication of mitochondria in insulin release and the exposure of pancreatic β-cells to hyperglycemia make them especially susceptible to oxidative stress and mitochondrial dysfunction. In fact, crosstalk between both mechanisms is related with alterations in glucose homeostasis and can lead to the diabetes-associated insulin-resistance status. In the present review, we discuss the current knowledge of the relationship between oxidative stress, mitochondria, ER stress, inflammation, and lipotoxicity in T2D.

## 1. Introduction

Diabetes is a disease considered to be a worldwide epidemic. The prevalence of diabetes among adults in 2017 was around 451 million cases, a number that is expected to rise to 693 million in 2045 [1]. Diabetes affects mainly developed countries, but its prevalence is increasing considerably in developing countries such as India and China [2]. Since it is a life-long condition, it represents an enormous economic burden on healthcare systems worldwide.

There are two main types of diabetes. Type 1 diabetes (T1D) is an autoimmune condition that causes the destruction of pancreatic β-cells and represents 5–10% of the total number of diabetes cases. On the other hand, type 2 diabetes (T2D) involves decreased insulin secretion by β-cells, or increased insulin resistance, and represents around 95% of all cases. T2D has been related to a high number of chronic comorbidities that can undermine the quality of life of patients and lead to the development of cardiovascular diseases (CVD). In fact, diabetes is associated with premature death, caused mainly by coronary artery disease, stroke, or renal dysfunction [2]. As already mentioned, T2D is related to insulin resistance and to several associated clinical complications, including obesity or atherosclerosis, and with a decrease in testosterone. T2D is a clinical syndrome described as a metabolic disturbance in which mitochondria play a key role. In fact, mitochondria are the main source of reactive oxygen species (ROS) and participate in redox homeostasis and other functions, such as apoptosis and Ca^2+^ metabolism [3,4] and ATP (adenosine triphosphate) production or heat generation. ROS can act as signaling molecules, but when their production is exacerbated, they induce mitochondrial dysfunction and a decrease in ATP production [5]. Mitochondrial dysfunction related to T2D, hyperglycemia, and insulin resistance has been described in different tissues, including skeletal muscle, kidney, lung, heart, and liver [6], as well as in circulating cells such as leukocytes [7,8].

## 2. Oxidative Stress in T2D

In both T1D and T2D, blood glucose levels are not regulated adequately, rising to abnormal levels for extended periods. This chronic hyperglycemia is characteristic of diabetes and the main attributor to the multiple complications associated with the disease. Although many aspects of the physiopathology of diabetes are still unclear, it is well established that oxidative stress plays a key role in the onset and development of this condition.

Oxidative stress is defined as an imbalance between the generation and elimination of ROS in favor of the formation of oxidants. ROS are oxygen-free radicals including hydroxyl radical (^•^OH), superoxide anion (O_2_^•−^), and peroxynitrite (ONOO^−^), among others. Other non-radical derivatives of oxygen are also considered ROS, such as hydrogen peroxide (H_2_O_2_), due to the ease with which it generates free radicals.

ROS are generated by a normal cell metabolism and perform important biological functions. While ROS are vital for life, due to their high chemical reactivity, they can damage macromolecules including lipids, proteins, and nucleic acids. For this reason, defense mechanisms are activated in cells to regulate the production of ROS and to avoid any oxidative injury. Most of the defense mechanisms against ROS are enzymes that scavenge excess ROS such as superoxide dismutases (SODs), catalase, peroxiredoxins, thioredoxins, and glutathione peroxidases.

**Figure 1:**
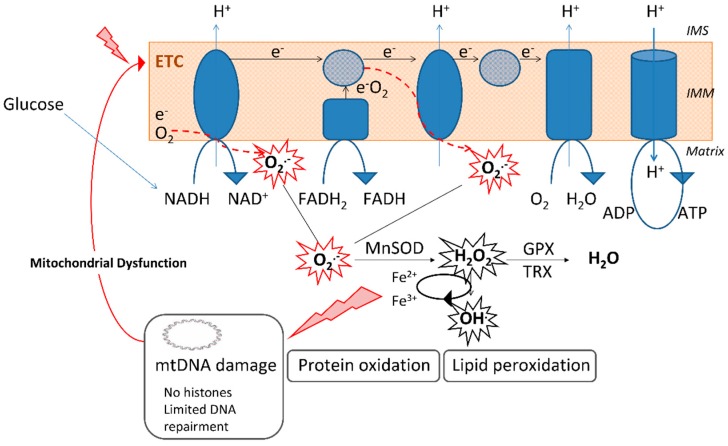
Mitochondrial superoxide (O_2_^•−^) generation by the electron transport chain (ETC) and the implication of the enzyme manganese superoxide dismutase (MnSOD), the only superoxide dismutase enzyme located in the mitochondrial matrix, in its detoxification. The elevated levels of O_2_^•−^ induce damage to macromolecules, including lipids, proteins, and nucleic acids, and promote mitochondrial dysfunction. Absence of histones in mitochondrial DNA (mtDNA) and limited DNA repair mechanisms make mitochondria highly susceptible to DNA damage induced by O_2_^•−^. ADP: Adenoxine diphosphate; ATP: Adenosine triphosphate; GPX: Glutathione peroxidase; H_2_O_2_: Hydrogen peroxide; IMM: Inner mitochondrial membrane; IMS: Intermembrane space; O_2_^•−^: Superoxide anion; OH^•^: Hydroxyl radical; TRX: Thioredoxin reductase.

Mitochondria are organelles that play a central role in the energetic metabolism, allowing energy to be obtained in the form of ATP through oxidative phosphorylation. In this process, NADH and FADH_2_ derived from the oxidation of nutrients are oxidized in the electron transport chain (ETC), thus generating ATP, ROS, and mainly O_2_^•−^. As a consequence, mitochondria are the major source of intracellular ROS and, under physiological conditions, exhibit antioxidant enzymes that maintain the cellular redox balance. The existence of a SOD that is particular to mitochondria—the manganese superoxide dismutase (MnSOD) —which deactivates O_2_^•−^— underlines the crucial role of mitochondria as a source of ROS and in maintaining them under homeostatic control (Figure 1).

Glucose is the main nutrient source of energy to fuel the ETC, generated in the form of NADH and FADH_2_. In light of this, it is not surprising that ROS are implicated in the physiopathology of diabetes. In fact, there is evidence that antioxidant enzymes are altered in type 2 diabetic patients [9], and multiple studies have observed a general oxidative stress status in patients with diabetes mellitus [10,11]. Furthermore, dysfunction of the mitochondrial ETC has been related to diabetes concerning mitochondrial diseases [12,13].

The mitochondrial genome encodes proteins that are part of the ETC and are essential for ATP production, and it is well established that mutations in the mitochondrial genome predispose subjects to diabetes [12,14,15]. For instance, a recent prospective study carried out in individuals with mitochondrial disease found a higher incidence of endocrine disorders such as diabetes mellitus [13]. A relationship between mitochondrial diseases and increased oxidative stress has also been described [16]. Due to the body of data demonstrating a connection between T2D and oxidative stress involving mitochondria, it has been proposed that mitochondria play a key role in the etiology of diabetes and the resulting ROS generation that is an important trigger of the consequences of the disease [17,18].

## 3. Oxidative Stress in Diabetes: Mitochondria and ER

As previously mentioned, mitochondria are implicated in the cell metabolism. Energy is obtained from mitochondria in the form of ATP through the process of oxidative phosphorylation. Therefore, mitochondria are a vital constituent of the cell, and one of the features of these organelles is the possession of their own genetic material, called mitochondrial DNA (mtDNA). Unlike the nuclear genome, mtDNA is not protected by histones, which renders it more exposed to ROS-induced damage. Besides, the capacity of mtDNA damage–repair mechanisms is limited compared to that of the nuclear DNA and polymerases involved in mtDNA replication; in addition, they are more prone to error and have a low frame of shift fidelity [19,20,21].

All of these features add up to a high ROS production by the mitochondria themselves, making mtDNA particularly susceptible to mutations induced by oxidative stress. Due to the fact that mtDNA encodes proteins that are part of the ETC, exposure to ROS increases the risk of ETC impairment, thus increasing oxidative stress even more.

**Figure 2:**
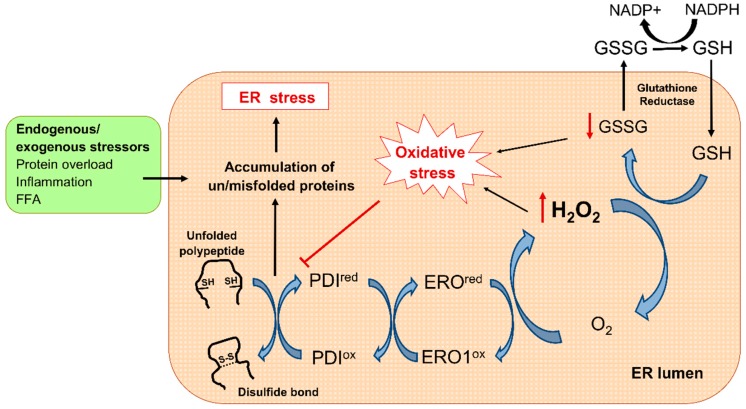
Oxidative protein machinery and ER stress. During disulfide bond formation, two electrons are transferred to the pair of cysteines in the polypeptide by the PDI active site. Thereafter, reduced PDI receive electrons from O_2_ through ERO1-mediated redox reaction, resulting in H_2_O_2_ formation. The GSH/GSSG system then recovers the redox status by scavenging H_2_O_2_. Several stimuli including increased protein synthesis demand overwhelm ER-folding capacity and disturb redox balance, leading to the accumulation of misfolded proteins and triggering ER stress. ER: Endoplasmic reticulum; ERO1: ER oxidoreductin 1; FFA: Free fatty acids; GSH: Glutathione; GSSG: Glutathione disulphide; NADPH: Nicotinamide adenine dinucleotide phosphate; PDI: Protein disulfide isomerase.

Although mitochondria are the main source of ROS production, it is important to point out that mitochondria are not the only organelle implicated in oxidative stress related to diabetes. Strong, growing evidence has brought to light that endoplasmic reticulum (ER) stress contributes to diabetes [22,23,24]. The ER is responsible for the correct folding of proteins into their functional three-dimensional conformation, whereby formation of disulfide bonds is a crucial process. To prevent and correct aberrant disulfide bonds, resident protein disulfide isomerases (PDI), endoplasmic reticulum oxidoreductin 1 (ERO1), and glutathione (GSH) cooperate as a chaperone-like assisted mechanism. This oxidative folding machinery produces large amounts of ROS and depletes GSH pool, thus contributing to redox imbalance [25]. In this sense, alterations in the ER lumen oxidizing environment can lead to illegitimate disulfide bond formation and accumulation of misfolded polypeptides, a condition known as ER stress [26]. In response to that, the oxidative folding machinery is hyperactivated to correct improper bonds, further producing ROS. In turn, the resulting hyperoxidizing ER lumen interrupts PDI proper function, thus contributing to the accumulation of misfolded proteins [27]. The consequences of this vicious cycle are a perpetuated status of oxidative and ER stress leading to disruption of ER function (Figure 2).

Both of the abovementioned organelles generate ROS: mitochondria, through the ETC, and ER, mainly via disulfide bond formation during the protein folding process. Both processes are also involved in the pathogenesis of diabetes, evident in the fact that the main consequence of T2D is high concentrations of circulating glucose. Glucose is used as fuel by mitochondria to obtain energy by ETC, a process of which ROS production is a by-product. High glucose concentrations can saturate antioxidant defenses and induce oxidative stress in mitochondria, but they can also induce insulin production. High ROS can overburden and saturate antioxidant mechanisms, thus leading to oxidative stress in the ER.

It is important to highlight that mtDNA is highly vulnerable to ROS-induced damage, which can result in mutations and the production of altered ETC proteins. This results in the malfunctioning of ETC and further generation of ROS, thereby accentuating oxidative stress in mitochondria. In turn, oxidative stress in the ER leads to the accumulation of unfolded proteins in the ER lumen, which, in turn, increases oxidative stress [28]. In this way, inside of each organelle, the oxidative stress generated by ROS production generates more oxidative stress, thus feeding a vicious cycle that interferes with the redox balance in different situations.

However, this overview of oxidative stress in mitochondria and the ER is somewhat simplistic. Far from being independent organelles, the two are tightly interconnected. A large body of evidence proves the existence of contact sites between both organelles, known as mitochondria-associated ER membranes (MAMs). Both organelles interact through MAMs to maintain cell homeostasis. MAMs allow the exchange of metabolites and ions between mitochondria and ER, and therefore each one is influenced by the oxidative state of the other. The ER lumen acts as a site for Ca^2+^ storage [29], which is a well-known mediator of ROS signaling [30]. In ER-stressed cells, Ca^2+^ is released from the ER and taken up by mitochondria, where it increases ROS in an indirect way. The activation of enzymes in the Krebs cycle and oxidative phosphorylation and inhibition of complex III of the ETC are additional mechanisms by which Ca^2+^ increases mitochondrial ROS production. In the regulation of redox homeostasis between mitochondria and ER, Ca^2+^ plays a crucial role as it evidences by the presence of the high concentration of IP3Rs (inositol 1,4,5 trisphosphate receptors), calcium-handling protein in MAMs [31].

In the context of mitochondrial function and ER stress, it is important to mention another physiological pathway—namely, autophagy [32]. Autophagy is a self-digestion process that degrades intracellular structures in response to stress, so it is not surprising that it is implicated in T2D. In fact, a large number of recent studies have described the protective function of autophagy in maintaining cellular homeostasis [33]. For instance, autophagy is activated in response to ER stress in order to recover ER function by degrading unfolded/misfolded protein aggregates and even portions of the dysfunctional ER itself. In this way, autophagy alleviates ER stress and reduces excess ER-ROS production, exerting as a regulatory mechanism for ER homeostasis. Moreover, mitochondrial ROS are considered central modulators of autophagy [34]. A specific form of autophagy (namely mitophagy) is activated and selectively eliminates damaged mitochondria, which are special contributors to mitochondrial ROS production. In this way, mitophagy is critical for maintaining a healthy population of mitochondria and reduces ROS accumulation. The crosstalk between autophagy and oxidative stress goes beyond the recovery of ER and mitochondrial function, since this pathway contributes to clearing the cells of all irreversibly oxidized macromolecules. In addition, growing evidence suggest that it also stimulates the transcriptional factor NRF2, which in turn promotes the expression of antioxidant and detoxifying genes [35].

Although autophagy is a well-studied process whose beneficial role in cells is evident, how exactly it influences T2D is unclear, and seems to depend on the type of tissue [36]. For instance, studies in skeletal muscle of diabetic patients have not reported alteration of autophagy [37], despite alterations in mitochondrial function and ROS being found [38]. Other studies have observed a decrease in proteins related to autophagy [39]. Since insulin inhibits autophagy [40], the down-regulation of autophagic proteins observed in skeletal muscle may be due to hyperinsulinemia in diabetic patients who have not yet developed insulin resistance [39]. In adipose tissue, an increase in autophagy has been reported [41], although it seems to depend on the type of adipose tissue and the presence of obesity and insulin resistance [42]. In the liver, autophagy seems to be suppressed in the presence of insulin resistance and hyperinsulinemia [43]. Overall, in muscle, adipose tissue, and liver—the major target tissues for insulin—it is not clear how autophagy is affected in T2D, although it would seem to be suppressed when diabetes and insulin resistance are present.

Another important function related to these processes is mitophagy. In fact, a decreased expression of mitophagic markers (selective mitochondrial autophagy) has been found in peripheral blood mononuclear cells of T2D patients, in contrast to the significant increase of the same markers observed in prediabetic subjects [44]. These results are in accordance with the progressive rise in oxidative stress and altered mitochondrial morphology associated with hyperglycemia in diabetic subjects. Until now, the literature has suggested that autophagy is suppressed by the chronic hyperglycemia and subsequent insulin resistance that define diabetes. The mitochondrial dysfunction and consequent mitochondrial oxidative stress characteristic of diabetes appear to be implicated in said suppression [44].

Beyond its implications in the cell pathology of diabetes, ROS has been closely related to its complications [6]. It is well known that hyperglycemia causes tissue damage, which renders diabetic patients highly susceptible to developing micro and macrovascular complications. O_2_^•−^, the most generated ROS during mitochondrial metabolism, is released in high amounts in vascular vessels as a consequence of hyperglycemia. O_2_^•−^ is deeply implicated in the pathogenesis of vascular complications through the inhibition of the GADPH (glyceraldehyde-3 phosphate dehydrogenase) enzyme. This enzyme participates in the glycolysis pathway, and its inhibition by O_2_^•−^ generates an increase in glycolytic intermediates such as glyceraldehyde-3-phosphate, fructose-6 phosphate and glucose [45,46].

Glyceraldehyde-3-phosphate is a metabolite of the advanced glycation end-products (AGE) pathway and the classic PKC pathway, two processes implicated in microvascular complications. The accumulation of fructose-6 phosphate increases the flux through the hexosamine pathway, which ultimately implies the expression of factors that are harmful for blood vessels. In addition, the inhibition of GADPH by O_2_^•−^ enhances the first glycolytic metabolite, glucose, thereby enhancing the polyol pathway, with the consequent consumption of the cofactor NADPH. In this context, antioxidant defenses are undermined, with a subsequent increase in ROS levels in vessels, thus promoting damage and vascular complications.

## 4. ROS and β-cells: Onset of Diabetes

ROS generation, mitochondrial dysfunction, ER stress, and alterations of autophagy are implicated in the development of T2D and are crucial to β-cell function. In these cells, ROS and disturbances caused by ROS constitute, at least in part, the onset of diabetes. The main function of β-cells is to maintain correct glucose homeostasis within the body by secreting insulin in response to increases in glucose concentration in blood. In brief, after the entrance of glucose mediated by GLUT2 (glucose transporter-2), glucose is phosphorylated to glucose-6-phospate and metabolized by glycolysis to produce pyruvate, NADH, and ATP. Pyruvate enters mitochondria to be oxidized by means of the tricarboxylic acid (TCA) cycle, leading to the production of more NADH. Finally, this NADH is metabolized to ATP production through the mitochondrial ETC. At the same time, hyperpolarization of the mitochondrial inner membrane can stimulate the mitochondrial membrane potential-dependent Ca^2+^ uniporter to increase mitochondrial Ca^2+^ and further stimulate TCA cycle activity. Eventually, there is an increase of ATP/ADP ratio, leading to closure of ATP-sensitive K^+^ channels, depolarization of the plasma membrane, opening of voltage-dependent Ca^2+^ channels and influx of Ca^2+^, thereby triggering exocytosis of insulin-containing granules.

**Figure 3:**
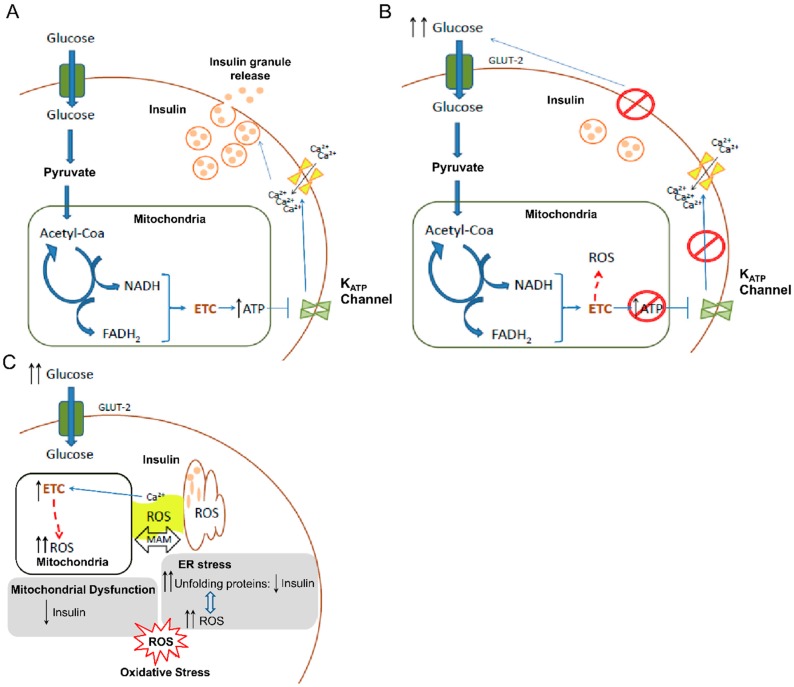
Cellular mechanisms in pancreatic β-cells involving mitochondrial ROS generation and their implication in insulin release and diabetes onset. (**A**) Mechanism of insulin release in β-cells in normal conditions. (**B**) Impaired insulin release by β-cells under hyperglycemic conditions. High glucose concentration in blood implies high ROS generation by mitochondria leading to alterations in insulin release. (**C**) Scheme of how hyperglycemia promotes ROS generation by ETC (electron transport chain) in mitochondria inducing oxidative stress. Oxidative stress is boosted by the ER stress as consequence of the accumulation of unfolded insulin peptides due to the enhanced demand of this hormone. In addition, the implication of Ca^2+^ ions from ER as a factor that increases ROS in mitochondria and the interconnection of both organelles through MAM (mitochondria-associated ER membranes) are depicted. ATP: Adenosine triphosphate; ER: Endoplasmic reticulum; GLUT2: Glucose transporter-2; ROS: Reactive oxygen species.

Importantly, it has been described that defective insulin secretion by β-cells underlies all forms of diabetes mellitus. Having seen how β-cells regulate the release of insulin, it is clear that mitochondria and, by extension, mitochondrial ROS generation, play an important role in β-cell function and T2D development. In addition, several characteristics of β-cells make them more susceptible to oxidative stress. First, β-cells are very active metabolically, but have weaker antioxidant defenses than other cells and tissues. In this sense, it has been shown that pancreatic islets express low activity of free radical detoxifying enzymes and redox-regulating enzymes [47,48,49]. Second, glucose is the main source of carbon in β-cells, with up to 80% being oxidized, a high percentage when compared with other cell types [50]. In addition, β-cells exhibit low lactate dehydrogenase, and so pyruvate is mostly metabolized by TCA to produce ATP in mitochondria [51]. Third, β-cells are exposed to higher glucose concentrations, as they cluster in islets that connect to the vasculature. Islets are perfused by a dense, specialized microcirculation and receive 10% of the pancreatic blood flow. The dense populations of capillaries surrounding islets are fenestrated, possessing a remarkable number of small pores that allow a greater exchange between circulation and cells. This structure enhances permeability, facilitating access to nutrients like glucose [52,53]. In addition, the receptor GLUT-2, through which β-cells uptake glucose, is distinctive for its high capacity and low affinity [54]. All of these features increase the velocity of glucose transport and the intracellular glucose concentration to detect glucose concentration in blood. However, these features mean that β-cells are exposed to high levels of glucose, and consequently to the damage induced by ROS derived from hyperglycemia (Figure 3).

Besides detecting glucose concentration in the blood, β-cells regulate concentration by secreting insulin, a critical regulatory protein of glucose metabolism. This requires a high demand of β-cells to synthesize insulin in response to increases in circulating glucose. Since ER is involved in protein synthesis, β-cells are particularly susceptible to ER stress through accumulation of unfolded proteins. Furthermore, a higher rate of protein synthesis increases disulfide bond formation for correct protein folding, thereby promoting the formation of ROS. In turn, ROS production promotes unfolded proteins and impairs ER function. As a result, more ROS are generated in what becomes a vicious cycle [55], with the consequent impairment of molecules, structures, and functions.

Overall, high mitochondrial ROS levels alter the mitochondria itself and the ER, thus increasing oxidative stress still further. Since both organelles are crucial for controlling blood glucose levels by β-cells, the hyperglycemic state becomes chronic, and diabetes develops.

Furthermore, as previously mentioned, an impairment of autophagy has been related to diabetes and how it is affected depends on the type of tissue or cells. Recently, autophagy has been the focus of great interest after being closely related to cellular homeostasis [56,57] and β-cell survival. An emerging body of evidence supports the role of autophagy in β-cells in the pathophysiology of T2D [32,58,59]. At first, it seems to exert a protective role by removing defective mitochondria and ER, preserving their integrity and preventing cell death by apoptosis, but the progressive dysfunction of β-cells renders them incapable of recovering, probably due to interference with the molecular pathway of mitophagy, which leads to their death [59,60,61].

**Figure 4:**
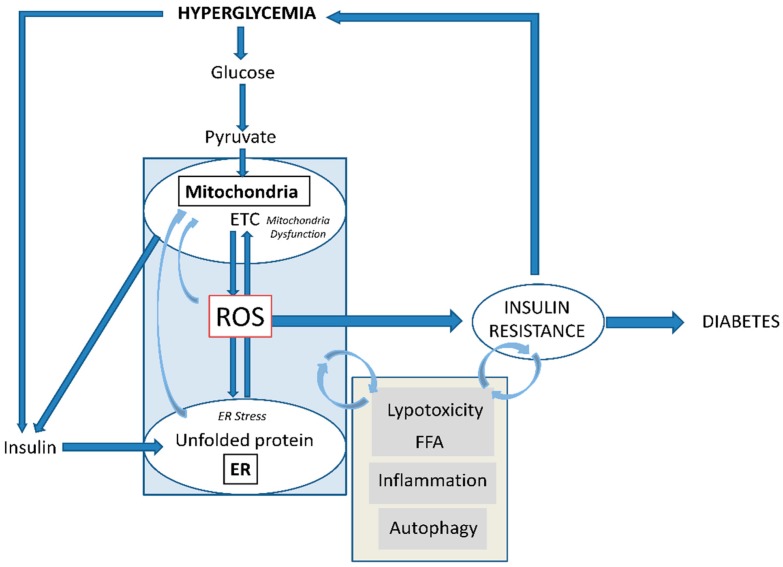
Development of insulin resistance and the relevant role of mitochondrial ROS generation. Frequent hyperglycemia condition promotes ROS production by mitochondria through the ETC. ER function and folding capacity is affected by mitochondrial ROS production. This process is especially important in pancreatic β-cells, in charge of insulin production and secretion. Initially, excess nutrient overload increases insulin synthesis demand. Perpetuated hyperglycemia and hyperinsulinemia progress to insulin resistance in peripheral tissues. This fact forces β-cells to produce more insulin promoting ER stress, in parallel with increased oxidative stress and mitochondrial dysfunction. Oxidative stress in mitochondria and ER stress feedback each other directly through ROS and also indirectly (curve arrow), aggravating the oxidative stress and promoting further insulin resistance. This situation can progress to β-cell failure and the impairment of insulin release, thus provoking the inability to control glucose levels in blood characteristic of T2D patients. Insulin resistance is also associated with processes such to inflammation, lipotoxicity, and autophagy impairment that make oxidative stress and insulin resistance characteristic of T2D worse. ETC: Electron transport chain; ER: Endoplasmic reticulum; FFA: Free fatty acids; ROS: Radical oxygen species.

Early on, an increase in nutrient input leads to β-cells proliferation and the stimulation of insulin release in response to low levels of ROS [62]. In addition, the activation of autophagy in these early stages seems to compensate starting cellular stress, resulting in a partial compensation of the nascent systemic insulin resistance, a situation known as β-cell compensatory mechanism. However, perpetuated state of oxidative stress, unfolded proteins accumulation and mitochondrial and ER dysfunction progressively impairs β-cells function and turns autophagic flux into the activation of apoptotic pathways, thus increasing their death rate [32]. Finally, the impairment of insulin secretion by altered β-cells is aggravated by the decline in β-cell mass (Figure 4).

## 5. Oxidative Stress and Inflammation: Crucial Role in Vascular Dysfunction

**Figure 5:**
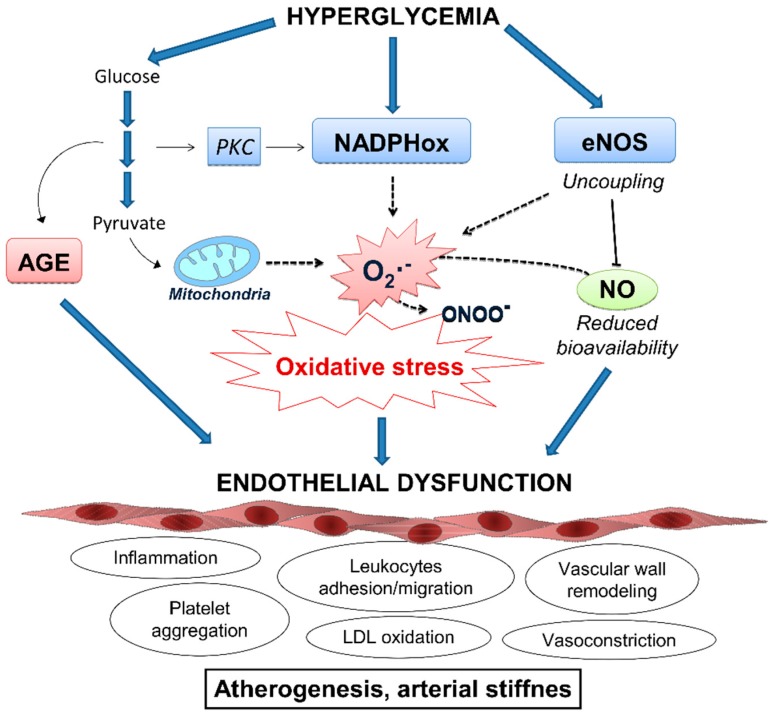
Mechanisms of ROS-induced endothelial dysfunction in response to hyperglycemia. Vascular damage caused by elevated glucose levels is mainly derived by an imbalance between ROS production and NO bioavailability in the endothelium and by the direct damaged caused by the accumulation of AGE. Resulting endothelial dysfunction is characterized by the activation of several deleterious mechanisms, including proinflammatory response, recruitment of leukocytes, accumulation of oxidized LDL particles and impaired vasodilatation, in the onset of cardiovascular complications. AGE: Advanced glycation end-products; eNOS: Endothelial nitric oxide synthase; LDL: Low density lipoprotein particles; NADPHox: Nicotinamide adenine dinucleotide phosphate oxidase; NO: Nitric oxide; O_2_^•−^: Superoxide anion; ONOO^−^: Peroxynitrite; PKC: Protein kinase C; RNS: Radical nitrogen species.

Vascular endothelial cells are one of the major targets of hyperglycemic damage due to their inability to modulate intracellular glucose concentration with respect to blood glucose levels, as they cannot prevent the glucose from entering when glucose concentrations in the bloodstream are elevated [63]. In this situation (during hyperglycemia), endothelial cells contain high levels of glucose and can suffer from pronounced oxidative stress. Both direct damage by AGE generated by glycation and damage indirectly caused by ROS during hyperglycemia can trigger an inflammatory response in the endothelium (Figure 5). Other mechanisms are also involved, such the deleterious action of AGE on their receptor (RAGE), which results in the production of ROS [64].

An important source of ROS in the diabetic vasculature is endothelial nitric oxide synthase (eNOS). Under physiological conditions this enzyme has beneficial effects by generating nitric oxide (NO) and producing vasodilatation. However, in the presence of high levels of ROS, NO rapidly reacts with O_2_^•−^, resulting in the formation of ONOO^−^—an oxidant agent—and further contributing to oxidative stress [65,66]. Furthermore, the augmentation of ROS levels reduces the availability of the eNOS co-factor (tetrahydrobiopterin), thus impairing the ability of eNOS to produce NO.

NADPH oxidase is another enzyme with an important role in ROS production at the vascular level. This enzyme catalyzes the production of O_2_^•−^ by transferring electrons to molecular O_2_ from NADPH or NADH. The production of O_2_^•−^ is the predominant function of NADPH oxidases [66,67], and not merely a by-product of the reaction, so these enzymes constitute the major source of O_2_^•−^ in vascular cells. In diabetes, the activity of NADPH oxidases is increased [68,69], contributing to the general oxidative stress state and inflammation of vascular tissue that are characteristics of the disease [69].

In T2D, the endothelium becomes dysfunctional, and an immune response is triggered by the invasion of immune cells such as neutrophils and macrophages. These immune cells generate ROS through a respiratory burst that alters the integrity of the endothelium [70]. All these factors produce ROS and oxidative stress in endothelial tissue, promoting the inflammatory status and impairment of the vascular endothelium. Furthermore, ROS promote inflammation by enhancing the levels of proinflammatory cytokines and the expression of cellular adhesion molecules and growth factors [71] in the onset of T2D-associated cardiovascular complications (Figure 5). In general, the inflammatory process contributes to insulin resistance and, consequently, to the progression of diabetes [72], which worsens the inflammation and creates a vicious cycle that further exacerbates the state of oxidative stress.

Beyond the effects it exerts in vascular tissue, inflammation seems to play a role in diabetes progression by directly affecting the integrity of the pancreas. The presence of macrophages in the pancreas has been reported in T2D, indicating an inflammatory process in pancreatic islets [73,74]. In this sense, inflammation causes pancreatic cell death through ROS [75]. ROS are probably involved in the onset of inflammation in pancreatic islets, and pancreatic cell death inevitably implies a loss of insulin secretion by β-cells, which in turn promotes insulin resistance.

## 6. T2D, Inflammation and Lipotoxicity

At the vascular level, inflammation plays an important role in microvascular complications (i.e., diabetic nephropathy, neuropathy, and retinopathy), and lipotoxicity is closely linked to macrovascular complications of diabetes, such as coronary artery disease, peripheral arterial disease, and stroke [76]. Therefore, it is clear that obesity is a risk factor in the pathogenesis of diabetes and insulin resistance. 

**Figure 6:**
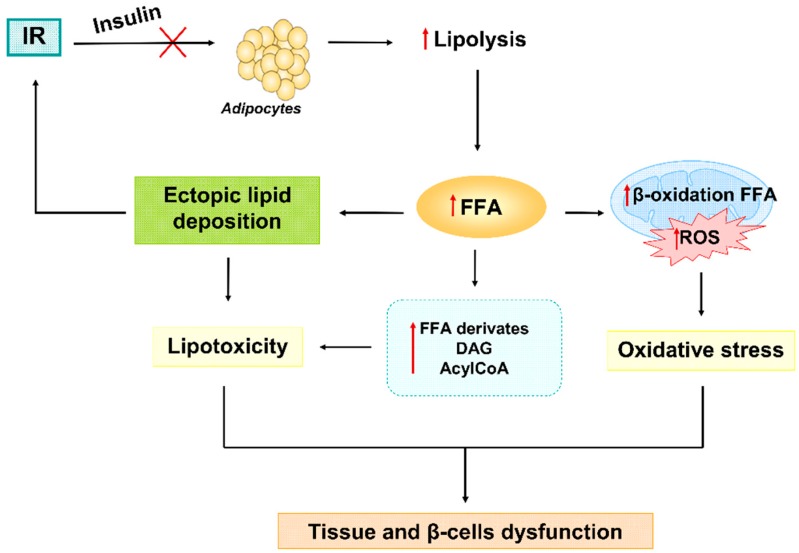
Under insulin resistance condition, adipocytes are not able to respond to insulin stimuli and downregulate lipolysis. In this way, an uncontrolled release of FFA causes ectopic lipid deposition in body tissues and an augment of lipid intermediates derivate from metabolism (DAG and AcylCoA), which together lead to lipotoxicity and further insulin resistance. On the other hand, FFA are not fully processed by mitochondria triggering incomplete β-oxidation of them, further ROS production and oxidative stress. Finally, lipotoxicity and oxidative stress they both concur to alterations in cells homeostasis and β-cells failure. AcylCoA: Acetyl coenzyme A; DAG: Dyacylglicerol; FFA: Free fatty acids; IR: Insulin resistance; ROS: Radical oxygen species.

In addition, one of the consequences of an inability to respond adequately to insulin is the increase of free fatty acids (FFA) in blood, especially due to the interruption of the antilipolytic effect of insulin on adipocytes. Fat cells respond to this by releasing large amounts of FFA to the bloodstream, thus initiating systemic lipotoxic effects including ectopic lipid deposition and subsequent further interruption of the insulin signaling. In this sense, lipotoxicity has recently emerged as an important contributor to insulin resistance. Lipotoxicity refers to cellular dysfunction, and injury to tissue is caused by excess FFA or toxic lipid intermediates like acylCoA, ceramide, and diacylglycerol (DAG) [77] (Figure 6). 

The association between ROS and lipotoxicity lies in the fact that they are oxidized in mitochondria by β-oxidation. The overload into the mitochondria because of increased FFA levels leads to an incomplete FFA oxidation, which generates an increase in ROS generation and toxic lipid intermediates (Figure 6). Due to altered mitochondria, the oxidation of FFA is also carried out in the ER, contributing to ER stress.

It is important to highlight that excess lipids are harmful in the case of saturated FFA, whereas mono and polyunsaturated FFA frequently exert antilipotoxic effects. The most abundant saturated FFA found in plasma is palmitic acid, which has been demonstrated to induce oxidative stress through β-oxidation in mitochondria and other pathways, leading to ER stress and perturbations in Ca^2+^ homeostasis. In this way, lipotoxicity induced by saturated FFA promotes mitochondrial dysfunction and aggravates oxidative stress, contributing in this way to insulin resistance [78]. In addition to oxidative stress, saturated FFA exacerbate insulin resistance status by causing inflammation [79]. These processes whereby FFA induces toxicity take place in several tissues throughout the organism, including β-cells. The effect of lipotoxicity on β-cells and its role in diabetes progression have made lipotoxicity a subject of increasing interest. Although high acute FFA concentrations in β-cells have been shown to promote β-cell proliferation, the chronic lipotoxic condition occurring in diabetes can promote the impairment of insulin secretion and eventually induce β-cell death. Finally, oxidative stress derived from mitochondria and ER dysfunction are also related to lipotoxicity in β-cells [80,81].

Strong, recent evidence has revealed a clear involvement of lipotoxicity in the progression of diabetes and oxidative stress. However, while diabetes is strongly related to obesity, not all obese subjects develop diabetes, and the toxic effect of lipid overload is generally manifested in coexistence with hyperglycemia [82,83]. Therefore, previous mitochondrial dysfunction and the existence of oxidative stress seem to condition the role of lipotoxicity in the development of diabetes, by which it aggravates insulin resistance and β-cell failure.

## 7. Targeting Oxidative Stress in T2D: Evidence on the Use of Antioxidants

Given the implication of oxidative stress in the onset and progression of diabetes, it is feasible that antioxidant strategies would be effective to prevent or treat diabetes. In this sense, vegetables and fruits are major sources of antioxidant compounds, and there exist a high number of studies reporting beneficial effects of different diets or foods in the prevention of diabetes [84,85,86,87,88,89]. Moreover, recent evidence has pointed out to carnosine—an antioxidant molecule found primarily in red meat—as a novel potential compound capable to diminish harmful effects of diabetes in health. In this sense, cellular studies have shown increased insulin secretion and enhanced glucose uptake derived from the ability of carnosine to scavenge oxidizing species [90]. Some clinical studies showed T2D and obese patients seem to benefit from carnosine supplementation by improving lipid profile, glucose management, and inflammation [91]. In addition, reduced oxidative stress after oral carnosine administration could be mediating a potential protective effect on cardiovascular [92] and renal function [93] in patients with T2D. However, most of published articles reporting benefits of carnosine on diabetes come from studies with animal models, so further clinical studies are required to delve deep into the potential use of this compound to treat metabolic disorders in humans.

In food, antioxidant compounds and other types of molecules form a complex matrix, and the beneficial effects of the former are usually manifested after regular consumption for prolonged periods. Until now, consumption of antioxidant compounds within the diet has been more of a preventive approach than a treatment, and it is clear that it is quite difficult to recover from diabetes—in the search for an effective drug to treat the disease, individual antioxidant compounds present in food have been widely studied [94,95,96,97,98,99,100,101].

Polyphenols and vitamins are the most well-known antioxidant with beneficial effects found in food. Many polyphenols and other phytochemicals have displayed antioxidant activity and positive effects on glucose homeostasis in preclinical studies. However, these beneficial effects are not always observed in clinical studies, possibly due to the fact that compounds that exert antioxidant activity through a direct ROS scavenger action are usually unstable. In addition, there are discordances between animal and human studies, which have been attributed to the concentration of the antioxidant compounds under study. In fact, the doses administrated in animal studies are usually higher than those used in humans. Overall, very few of the antioxidant phytochemicals tested have demonstrated a real benefit in diabetes treatment, and subsequent research into the underlying mechanisms has usually revealed processes that go beyond ROS scavenger activity. As explained above, diabetes implies a general condition of oxidative stress in the whole body, with the implication of numerous factors that feed back to each other and increase oxidative stress in what is a spiraling vicious cycle. When this situation is established, strategies based on antioxidant-free radical scavenging are not enough to alleviate oxidative stress. For this reason, the compounds showing real benefits in diabetes usually act through pathways by which antioxidant factors and enzymes are restored, pathways of ROS generation are blocked, and gene expression is altered [90,102].

In addition, these antioxidant compounds frequently display actions on other factors implicated in diabetes, like inflammation, autophagy or β-cell proliferation [96,101,103,104,105]. A clear example is resveratrol, a polyphenol whose high antioxidant activity has been shown to prevent and protect against diabetes and its complications, as well as improving glucose homeostasis [106,107,108].

Vitamins C and E, micronutrients and important constituents of our diet, have been widely studied for their use in diabetes prevention and treatment due to their antioxidant potential. Indeed, there is some controversy surrounding the antioxidant versus prooxidant activity of vitamins and their possible toxicity. However, the most recent studies, reviews, and meta-analyses on this subject conclude that these compounds can be effective in increasing antioxidant capacity and preventing or controlling diabetes and its complications [109,110,111]. The combination of antioxidant compounds is also a strategy to potentiate antioxidant effects [112]. In this context, the use of supplements containing natural compounds with antioxidant properties like polyphenols is under evaluation [113,114].

Another strategy to improve the efficacy of antioxidant compounds is to modify the chemical structure of known antioxidant compounds in order to increase their stability and antioxidant efficacy [115,116]. A combination of an antioxidant compound and antidiabetic drugs has also been assessed as an approach for treating diabetes [117].

It has been demonstrated that the effectiveness of several antidiabetic drugs is due to their antioxidant activity. For example, the main action of thiazolidinediones is exerted through peroxisome proliferator-activated receptor gamma (PPARγ). The activation of these receptors increases the transcription of a number of genes, some of which include antioxidant enzymes such as SOD and catalase [118,119,120]. Furthermore, thiazolidinediones inhibit intracellular-free radical overproduction through iNOS and NF-κB pathways implicated in ROS generation during diabetes [121]. The sulphonylurea gliclazide also expresses activity as a free-radical scavenger [122,123].

Another drug used in the treatment of T2D is metformin, whose antidiabetic effect has been shown to take place through a reduction in hepatic glucose production. Moreover, the antioxidant action of metformin has also been reported through reductions in ROS production in both in vitro and in vivo studies. For example, it inhibits mitochondrial respiration by diminishing mitochondrial complex I activity, thereby decreasing mitochondrial respiration [124,125]. In addition, it enhances NO release and reduces nitroxidative stress [124,126,127,128,129,130]. These findings support the benefits of metformin in diabetes beyond its effects on glucose levels.

Some of the newest antidiabetic drugs that have been placed on the market also have antioxidant effects, such as Glucagon-like peptide-1 (GLP-1) agonists, and dipeptidyl-peptidase-4 (DPP-4) inhibitors. Their antidiabetic effects are based on a decrease in blood glucose produced through an increase in insulin secretion and an inhibition of glucagon release. Besides this mechanism of action, GLP-1 has recently been demonstrated to induce antioxidant effects by enhancing the expression of antioxidant enzymes and activating NRF2 [131,132,133]. By extension, both GLP-1 agonists and DPP-4 inhibitors are found to exhibit antioxidant activity. This effect has been proved in vivo for some DPP-4 inhibitors —linagliptin, sitagliptin and alogliptin [134,135,136]— and in vitro for teneligliptin [137]. The GLP-1 agonists exenatide [138,139,140], and lixisenatide [141] also showed antioxidant effects.

Taking into account that mitochondria are the origin of the ROS causing hyperglycemia, multiple strategies have been developed to specifically target them [142,143]. Mitochondria are surrounded by a double-membrane system: an outer membrane separated from the inner membrane by an intermembrane space with metabolite carriers. In addition, the inner membrane has membrane potential (negative on the inside) and a pH gradient (basic on the inside). These properties of mitochondria are relevant for drug targeting, and so structures with a lipophilic and cationic nature have been tested to measure their accumulation inside the organelle. Such is the case of lipophilic cations that carry the antioxidant MitoQ and cationic plastoquinone derivatives, like SKQ1, which is a rechargeable antioxidant, in a similar way to MitoQ. These strategies have shown beneficial effects in metabolic pathologies and represent a promising approach to pathologies with oxidative components [142,143,144].

The use of hydrophobic and positively charged peptides that carry antioxidant agents to mitochondria is another way of selectively delivering elements into the mitochondria. The specific uncoupling of ETC in order to prevent formation of ATP and ROS constitutes another approach to combat oxidative stress. Furthermore, the use of nanoparticles is being studied as an alternative delivering method [142,143,145,146,147].

Thanks to the expansion in our knowledge about the physiopathology of T2D, oxidative stress has been confirmed as an important player in the onset and progression of the disease. In this way, targeting oxidative stress with antioxidant compounds has emerged as a relevant field of research for the identification and development of new drugs for T2D prevention and treatment.

## 8. Conclusions

T2D is a highly prevalent chronic metabolic disorder. Despite being an important focus of investigation, there is still a much to learn about its physiopathology, as it is not clear what triggers it, and multiple factors seem to be implicated. Extensive research continues to unravel the molecular causes of diabetes development. In this context, oxidative stress has been widely recognized as a main player in the development of the disease and a driver of diabetic complications. A large number of scientific articles have provided a deeper understanding of the physiopathology of diabetes. Hence, oxidative stress is not only a consequence of the disease but also plays a role in its onset.

Oxidative stress is the outcome of the overgeneration of ROS that are not neutralized by scavenging or detoxification pathways, thus resulting in a redox imbalance. This review attempts to offer a brief overview of the role of ROS and oxidative stress as triggers of diabetes. We have focused on the most important processes implicated in ROS generation during the onset and progression of diabetes in order to understand how ROS are involved in the disease. In this context, it is relevant to mention that T2D is characterized by insulin resistance and that insulin release is stimulated mainly by hyperglycemia. When β-cells are frequently exposed to hyperglycemia, the glucose metabolism is enhanced and there is an increase of ROS derived from mitochondrial ETC. The increase in mitochondrial ROS induces and/or worsens ER stress, an event that is prone to occur in β-cells due to the high demand for the synthesis and secretion of insulin in response to hyperglycemia. In addition, ER stress in itself increases ROS generation and glucose metabolism, leading the mitochondria to produce more ROS. Therefore, the ROS generated in mitochondria induce ER stress, and ER stress promotes ROS generation in mitochondria, resulting in a vicious cycle that produces a general condition of spiraling oxidative stress. The cycle continues, and an oxidative stress state is established in β-cells, which become unable to respond adequately to hyperglycemia. In addition, ROS damage cellular components, including lipids, protein, and DNA, and trigger transcriptional changes that promote insulin resistance. In this way, insulin resistance is initiated, which leads to chronic hyperglycemia and the generation of further ROS, with the ultimate result being β- cell dysfunction.

Alteration of molecular and cellular components by oxidative stress effects cellular mechanisms such as autophagy or inflammation. These processes are altered or disrupted, which contributes to a further increase in oxidative stress and insulin resistance.

One of the many consequences of insulin resistance is the increase of FFA, which generates lipotoxicity. High FFA concentrations promote an increase of ROS by stimulating mitochondrial metabolism and ER activity. Lipotoxicity has recently been recognized as playing an important role in the dysfunction and death of β-cells.

Beyond β-cells in the pancreas, several cell types in the whole body are affected by hyperglycemia and the consequent oxidative stress. The cells of vessels are more exposed to hyperglycemia, so they are more seriously damaged. The inflammatory response to the damage induced by hyperglycemia and ROS becomes chronic as diabetes progresses and constitutes the main cause of vascular complications. Along with the inflammatory component, there is an increase of FFA in the blood due to insulin resistance. The vascular state deteriorates with high concentrations of FFA, and the outcome is macrovascular complications such as coronary artery disease, peripheral arterial disease, and stroke, which often lead to premature death of the diabetic patient.

In view of the important role of oxidative stress in T2D, the use of antioxidant agents represents a promising therapeutic strategy. Potent antioxidant agents seem to have beneficial effects by preventing or ameliorating vascular complications. However, although oxidative stress clearly plays an important role, diabetes is a multifactorial disease involving a plethora of mechanisms. When diabetes is established, a multitude of interconnected events takes place, which makes it very hard to treat the disease. To manage T2D, it would appear to be necessary to target several pathways at the same time. The antioxidant approach is clearly one of them, as evidence shows that the antioxidant effect of therapeutic drugs currently used in diabetes underlies their antidiabetic efficacy.
